# Biocide effects of volatile organic compounds produced by potential biocontrol rhizobacteria on *Sclerotinia sclerotiorum*

**DOI:** 10.3389/fmicb.2015.01056

**Published:** 2015-10-06

**Authors:** Annalisa Giorgio, Angelo De Stradis, Pietro Lo Cantore, Nicola S. Iacobellis

**Affiliations:** ^1^Scuola di Scienze Agrarie, Forestali, Alimentari ed Ambientali, Università degli Studi della BasilicataPotenza, Italy; ^2^Istituto per la Protezione Sostenibile delle Piante, Consiglio Nazionale delle RicercheBari, Italy

**Keywords:** rhizobacteria, phytopathogenic fungi, *Sclerotinia sclerotiorum*, volatile organic compounds, transmission electron microscope, ultrastructures, hemolysis

## Abstract

Six rhizobacteria isolated from common bean and able to protect bean plants from the common bacterial blight (CBB) causal agent, were *in vitro* evaluated for their potential antifungal effects toward different plant pathogenic fungi, mostly soil-borne. By dual culture assays, the above bacteria resulted producing diffusible and volatile metabolites which inhibited the growth of the majority of the pathogens under study. In particular, the latter substances highly affected the mycelium growth of *Sclerotinia sclerotiorum* strains, one of which was selected for further studies either on mycelium or sclerotia. Gas chromatographic analysis of the bacterial volatiles led to the identification of an array of volatile organic compounds (VOCs). Time course studies showed the modification of the VOCs profile along a period of 5 days. In order to evaluate the single detected VOC effects on fungal growth, some of the pure compounds were tested on *S. sclerotiorum* mycelium and their minimal inhibitory quantities were determined. Similarly, the minimal inhibitory quantities on sclerotia germination were also defined. Moreover, observations by light and transmission electron microscopes highlighted hyphae cytoplasm granulation and ultrastructural alterations at cell organelles, mostly membranes, mitochondria, and endoplasmic reticulum. The membranes appeared one of the primary targets of bacterial volatiles, as confirmed by hemolytic activity observed for the majority of pure VOCs. However, of interest is the alteration observed on mitochondria as well.

## Introduction

In recent years biological control, through the application of antagonistic microorganisms from the rhizosphere (Paulitz and Bélanger, 2001; Minuto et al., 2006), has raised great research interest as a possible alternative for plant protection because it seems to be eco-friendly and offers a secure long-term protection of the crops (Fernando et al., 2005). Bacterial antagonists can negatively affect the growth of plant pathogens by several mechanisms such as the excretion of antifungal metabolites e.g., antibiotics, toxins, and bio-surfactants (Raaijmakers et al., 2002). Recently, it was demonstrated that volatile organic compounds (VOCs) of soil bacteria can influence the growth of phytopathogenic fungi as well (Alström, 2001; Wheatley, 2002).

The VOCs are generally lipophilic substances with high vapor pressure which freely pass through biological membranes and are released into the atmosphere or in the soil where the producers are (Pichersky et al., 2006). Therefore, the volatiles produced by soil bacteria may have a role above the ground, but also within the soil. Some VOCs may act as signal substances for inter- and intra- organisms communication as well as between cells of the same organism (Kai et al., 2009). Over the past years, attention has focused on the study of the production of VOCs by microorganisms as a weapon of defense against pathogenic fungi (Mackie and Wheatley, 1999; Strobel et al., 2001; Fernando et al., 2005; Gu et al., 2007; Zou et al., 2007; Liu et al., 2008; Wan et al., 2008; Arrebola et al., 2010). However, research on VOCs, in the interactions between potential biological control agents (BCAs), plant pathogens and host or non-host plants, as well as the biotic and abiotic factors that influence these relationships is still in its infancy (Campos et al., 2010). Therefore, in this regard, continuous research is needed.

In our previous studies six rhizobacteria, isolated from bean in southern Italy, resulted to protect bean plants from *Xanthomonas axonopodis* pv. *phaseoli* var. *fuscans*, the causal agent of common bacterial blight (CBB). Then, three of them were demonstrated triggering induced systemic resistance (Van Loon, 2007) in the plant model *Arabidopsis thaliana* toward *Xanthomonas campestris* pv. *armoraciae* either when they were inoculated in soil or via bacterial volatiles (Giorgio et al., 2014). Furthermore, rhizobacteria showed some typical features of plant growth promoting rhizobacteria (Giorgio et al., 2015), hence they appeared as potential BCAs. As a consequence, it seemed interesting to investigate about the activity of rhizobacteria against other bean pathogens with a lifestyle different from the bacterial pathogens ones. Indeed, the aim of the present work was to evaluate the possible antifungal properties of the mentioned bacterial potential BCAs toward several, mostly soil-borne, phytopathogenic fungi, focusing the attention on the biological effects of bacterial volatiles. Among the pathogenic fungi used in this study, strains of *Sclerotinia sclerotiorum*, a cosmopolitan necrotrophic fungal pathogen characterized by a broad host range (Purdy, 1979), resulted highly sensitive to both diffusible and volatile antimicrobial substances and, for that reason, one strain of the above pathogen was selected for further studies. Here we report *in vitro* fungal growth inhibition by diffusible and volatile substances produced by six bean rhizobacteria. In particular, some pure VOCs, identified by GC-MS, were evaluated, in comparison to the natural volatile blends, for their specific antifungal activity on *S. sclerotiorum* mycelium and sclerotia germination, for hemolytic activities and, for their effects at cellular and ultrastructural levels on the pathogen mycelium in order to figure out the probable action mechanisms of the above mentioned volatiles.

## Material and methods

### Bacteria and fungi growth conditions

Bacteria were isolated from the rhizosphere of bean plants in the National Park Agri valley, in southern Italy and preliminarily identified on the basis of their nutritional profiles by BIOLOG system (Biolog, Inc. Hayward, CA, USA) and partial 16S rDNA sequencing. Three of them showed an elevated sequence homology with strain NFM421 of *Pseudomonas brassicacearum* (Ortet et al., 2011) (USB2101: ID HE981747; USB2102: ID HE981748; USB2104: ID HE981749, EMBL, 2013); two isolates resulted similar to the strain W619 of *P. putida* (Copeland et al., unpublished data) (USB2105: ID HE981750; USB2106: ID HE981751, EMBL, 2013) and one isolate revealed high similarity with strains DSM 319 and QMB 1551 (Eppinger et al., 2011) of *Bacillus megaterium* (USB2103: ID HE981752, EMBL, 2013). Since the above identification is not yet definitive along this manuscript the five fluorescent pseudomonads have been reported as *Pseudomonas* spp. and the Gram positive bacterium as *Bacillus* spp. The rhizobacteria were grown on King's B agar (KBA) (King et al., 1954) at 25°C for 48 h. For short term storage, bacteria were grown on glycerol nutrient agar (GNA) slants and stored at 4°C (Lelliott and Stead, 1987). For long-term storage, bacteria were lyophilized or maintained at −80°C in 30% glycerol. They were previously phenotypically characterized showing some typical biocontrol traits (Giorgio et al., 2015) (Supplementary Table 1).

Fungal strains used in the present work (Table 1) were grown on potato dextrose agar (PDA) for 5 days at 25°C and maintained on the same medium at 4°C.

**Table 1:** **Plant pathogenic fungi used in this study**.

**Fungal strains**	**Plant host**	**Geographical origin**
*Botrytis cinerea* USB-F1131	Grape	Italy
*B. cinerea* USB-F1636	Pepper	Italy
*Fusarium equiseti* USB-F2014	Soil	Italy
*F. oxysporum* USB-F1	Tomato	Greece
*F. oxysporum* USB-F111	Strawberry	Italy
*F. solani* USB-F607	Bean	Italy
*Macrophomina phaseolina* USB-F918	Pepper	Italy
*Phytophthora cactorum* USB-F876	Chestnut	Italy
*P. cactorum* USB-F1168	Strawberry	Italy
*P. nicotianae* USB-F172	Chestnut	Italy
*Pythium ultimum* USB-F2	Potato	Greece
*Rhizoctonia solani* USB-F3	Pepper	Greece
*Rosellinia necatrix* USB-F587	Cherry	Italy
*Sclerotinia sclerotiorum* USB-F593	Bean	Italy
*S. sclerotiorum* USB-F853	Cantaloupe	Italy
*Verticillium dahliae* USB-F464	Chicory	Italy
*V. dahliae* ITM-1910	Tomato	Italy

USB, Università degli Studi della Basilicata, Potenza, Italy; ITM, Istituto Tossine e Micotossine, Consiglio Nazionale delle Ricerche, Bari, Italy.

### Bacteria production of bioactive substances

#### Diffusible substances

Dual culture assays were performed in order to test the possible antagonistic activity of rhizobacteria toward 17 strains of phytopathogenic fungi of different origin. In particular, two fungal plugs (5 mm ø), from a 5 days culture on PDA, were taken from the actively growing fungal colony edge and placed, to the opposite sides, on the surface of 20 ml KBA in Petri dishes. Then, a droplet of 50 μl of bacterial suspension (10^8^ CFU ml^−1^), from an overnight culture on KBA, was inoculated in the middle of the Petri dish, between the two fungal plugs. The assay was performed also on minimal medium agar (MMA) (Lavermicocca et al., 1997) and PDA. Controls were prepared in a similar manner without bacterial suspension in the middle of plates. After 5 days incubation at 25°C the diameter of fungal colony was measured. For the determination of the inhibitory effect of the bacterial isolates on pathogenic fungi the inhibition rate (IR%) was calculated according to the following formula: IR% = 100 × [(C-B)/C], where C is the diameter of the control fungal mycelium and B the diameter of the fungal mycelium grown in the presence of the antagonistic bacteria. The experiments were performed three times with three replicates.

#### Volatiles compounds

Rhizobacteria were tested for their ability to produce volatile substances inhibiting fungal growth using the double plate technique. One hundred microliters of bacterial suspensions, prepared as described above, were spread on KBA in Petri dishes. Three plugs of mycelium (5 mm ø) were placed on PDA surface for each plate. Petri dishes containing fungal mycelium plugs were then placed inverted over the KBA plates inoculated with the bacteria. Each pair of plates was sealed with Parafilm® to prevent the leak of bacterial volatiles compounds. The plates were incubated at 25°C for 5 days. Control sets were prepared in a similar manner, but without bacteria. The diameter of the fungal colony was measured after the incubation period. The assays were performed three times with three replicates.

In order to verify whether bacterial volatiles could have fungicidal or fungistatic action, plugs of fungal mycelium of the strain USB-F593 of *S. sclerotiorum* exposed to volatiles substances for 5 days were taken and re-inoculated on fresh PDA incubated at 25°C for 5 days. The assay was performed three times with three replicates.

With the aim of testing bacterial volatiles effects on *S. sclerotiorum* sclerotia, three sectors Petri dishes were used filling two out three parts with PDA, on which sclerotia (one sclerotium per sector) were placed, and the third one was inoculated with a droplet of 50 μl of bacterial suspension (10^8^ CFU ml^−1^) on KBA. Sclerotia were produced inoculating 100 ml potato dextrose broth (PDB) with three agar plugs of *S. sclerotiorum* in 250 ml flasks. After incubation at 20°C for 4 weeks, the flasks were incubated at 4°C for further 4 weeks to condition the sclerotia (Dillard et al., 1995). The sclerotia of 2–4 mm ø size were selected, then washed, dried overnight in a stream of sterile air, and used for the assay. The radial growth of the mycelium growing from the sclerotia was measured after 5 days incubation at 25°C. The experiment was performed three times with three replicates per treatment. In order to verify sclerotia viability after bacterial volatiles exposure for 5 days, sclerotia were re-inoculated on fresh PDA and incubated at 25°C for 5 days. The assay was performed three times with three replicates.

All data of bioassays described above were statistically analysed for the determination of standard errors, for ANOVA and *P* was calculated by *F*-test of Fisher-Snedecor. All statistical analysis were carried out using the SPSS version 17.0 software program package (SPSS Inc., Chicago, IL).

### GC-MS analysis

One hundred microliters of bacterial suspensions (10^8^ CFU ml^−1^) were inoculated on the surface of KBA slants in glass tubes equipped with silicone septa (Supelco 12345-U) caps and incubated for 5 days at 25°C. Volatile compounds were collected from the head space of the tubes by solid phase micro-extraction (SPME) technique (Zhang and Pawliszyn, 1993; Strobel et al., 2001). For the purpose SPME fiber coated with 100 μm of a phase of polidimetilsiloxane (Supelco 57 300-U, mounted on a support 57 330 Supelco) was conditioned for 1 h at 250°C in a stream of helium and then introduced for 20 min into the head space of the tube containing bacterial suspension. Then the fiber was introduced into the injection port of a gas chromatograph HP6890 equipped with a capillary column Phenomenex Zebron ZB-5 MS (30 m × 0.25 mm ID × 0.25 mm film thickness). HP5973 mass spectrometer (mass range: 15–300 amu, scan speed: 1.9 scans s^−1^, the voltage EM: 1435) was used as mass selective detector. Helium was used as carrier gas (0.8 ml min^−1^ flow rate) and the desorption time was 1 min. The injection port was maintained at 250°C while the detector at 230°C. The oven was maintained at a temperature of 40°C for 2 min to increase up to 250°C (8°C min^−1^). The run method was set at 33 min.

All peaks were identified by their mass spectra in comparison with the spectra present in Wiley6N and NIST98 databases.

The isolates USB2103, USB2104, and USB2105 attributable to strains of *B. megaterium, P. brassicacearum*, and *P. putida*, respectively, were chosen to perform a time course GC analysis at 1, 3, 5 days incubation. VOCs collection, analysis and identification have been achieved as described above.

All the GC-MS analysis were performed three times.

### Effects of pure VOCs on *Sclerotinia sclerotiorum* mycelium and sclerotia

Double plate technique and three sectors Petri dishes experiments described above for mycelia and sclerotia, respectively, were used, with some modifications, to carry out pure VOCs assay, in order to verify individual VOC action on the strain USB-F593 of *S. sclerotiorum*. In particular, two plugs of mycelium, grown on PDA at 25°C for 5 days, were placed in Petri dishes on PDA. A watch glass, previously sterilized (121°C for 20 min), was placed on the lid of each plate and filled with 100 μl of pure VOC. Then each plate was inverted on its cover containing the watch glass and the Petri dish was Parafilm® sealed to prevent leakage of VOCs. For sclerotia assay, the watch glass was placed in one out three sectors of Petri dishes; while sclerotia were put as already described for natural bacterial volatiles exposure.

Petri dishes were incubated at 25°C for 5 days.

Starting from a volume of pure VOCs of 100 μl, 1:1 serial and, when necessary, intermediate dilutions were made in order to define the fungal growth minimal inhibitory quantity (MIQ) (expressed in milligrams) for mycelium growing either from the fungal plug or from the germinated sclerotia. Pure substances were diluted in water, DMSO or methanol depending on their solubility. The assay was carried out as described above using watch glasses and Petri dishes were incubated at 25°C for 5 days.

### Hemolytic activity of pure VOCs

According to the bacterial volatiles profile results obtained via GC-MS analysis, some VOCs, chosen on the basis of their systematic detection and availability on the market (Sigma-Aldrich, Milan, Italy), were individually used to investigate their possible activity on biological membranes via hemolytic assay according to Lo Cantore et al. (2006).

One drop of 2 μl of pure VOCs and their dilutions, in solvents such as water, DMSO or methanol, depending on their solubility, were placed, equally distanced, on blood agar base medium. After 48 h at 25°C, the minimal hemolytic quantity (MHQ), expressed in milligrams, which causes an evident hemolytic spot in correspondence of the application point was recorded.

The assay was performed three times with three replications.

### Microsopic observations and ultrastructural studies

In order to evaluate bacterial volatiles action on fungal cell structures, samples of mycelium were collected from the tip of growing mycelium after 5 days of exposure to bacterial volatiles as well as to pure VOCs. Mycelium was analyzed by light microscope (Zeiss Axioskop 40, Carl Zeiss Microscopy, Thornwood, NY, United States), at a resolution 100x. The images were captured with a digital camera Olympus C-7070 Imaging software by Delta System IAS2000.

For thin sectioning, mycelium fragments were excised from the tip of growing mycelium and processed for transmission electron microscopy (TEM) analysis according to embedding standard procedures (Martelli and Russo, 1984). Briefly, mycelium was fixed in 4% glutaraldehyde in 0.05 M potassium phosphate buffer (pH 7.2) for 2 h and then it was post-fixed at 4°C in 1% osmium tetroxide in the same buffer for 2 h. Overnight bulk staining in 0.5% aqueous uranyl acetate, dehydration in graded ethanol dilutions, and embedding in TAAB Spurr resin followed. Thin sections were stained with lead citrate before observations with a Philips Morgagni 282 D (FEI Company, Hillsboro, OR) transmission electron microscope at 60 KV accelerating voltage.

## Results

### Bacterial production of bioactive substances

#### Diffusible substances

In dual culture assays on KBA rhizobacterial diffusible substances resulted to inhibit, albeit with different effectiveness, almost all the phytopathogenic fungi under study (Table 2). In general, the *Pseudomonas* spp. isolates USB2101 and USB2104 resulted more active in the fungi growth inhibition, whereas the isolate USB2103 of *Bacillus* spp. was the less effective (Table 2). Moreover, the strains USB-F1131 of *Botrytis cinerea*, USB-F876, and USB-F1168 of *Phytophthora cactorum* and USB-F587 of *Rosellinia necatrix* were the most sensitive fungi to bacteria diffusible metabolites action; on the contrary, strain USB-F2014 of *Fusarium equiseti* was the less sensitive to the above mentioned substances (Table 2). A similar antifungal activity, though reduced, was observed when bacteria were grown on MMA (data not shown). On this latter medium only the isolate USB2103 *Bacillus* spp. showed an higher activity when compared to its own action on KBA. In contrast, bacteria grown on PDA presented a very low or completely lacked inhibitory activity (data not shown).

**Table 2:** **Growth inhibition, determined in dual plate assays, of phytopathogenic fungi exposed to diffusible substances produced by six bacteria isolated from bean rhizosphere**.

**Phytopathogenic fungal strains^a^**	**Treatments^b^^,^^c^**						
	**Control**	**USB2101**	**USB2102**	**USB2103**	**USB2104**	**USB2105**	**USB2106**
*Botrytis cinerea* USB-F1131	1.6±0.04	0^*^	1.1±0.03^*^	1.5±0.03	0^*^	0.8±0.05^*^	1.1±0.03^*^
*B. cinerea* USB-F1636	1.6±0.06	1.1±0.03^*^	1.1±0.03^*^	1.5±0.02	0.8±0.06^*^	1.3±0.03^*^	1.3±0.04^*^
*Fusarum equiseti* USB-F2014	2.8±0.03	2.0±0.06^*^	2.6±0.03^**^	2.6±0.02	2.7±0.03	2.7±0.05	2.7±0.04
*F. oxysporum* USB-F1	2.8±0.04	2.1±0.03^*^	2.3±0.04^*^	2.6±0.04^**^	2.1±0.04^*^	2.4±0.04^*^	2.3±0.03^*^
*F. oxysporum* USB-F111	5.8±0.04	2.7±0.03^*^	3.4±0.05^*^	5.6±0.04^**^	4.7±0.06^*^	4.4±0.04^*^	4.7±0.05^*^
*F. solani* USB-F607	5.6±0.03	3.4±0.05^*^	3.2±0.02^*^	4.6±0.05^*^	3.3±0.06^*^	4.5±0.07^*^	4.6±0.06^*^
*Macrophomina phaseolina* USB-F918	5.9±0.04	3.1±0.03^*^	3.3±0.04^*^	5.8±0.03	3.4±0.04^*^	4.2±0.04^*^	4.3±0.04^*^
*Phytophthora cactorum* USB-F876	1.7±0.02	0.6±0.06^*^	1.1±0.03^*^	1.8±0.05	1.1±0.03^*^	1.3±0.04^*^	1.3±0.05^*^
*P. cactorum* USB-F1168	1.8±0.03	0.5±0.03^*^	1.1±0.03^*^	1.8±0.05	1.1±0.04^*^	1.3±0.05^*^	1.3±0.06^*^
*P. nicotianae* USB-F172	2.2±0.07	2.1±0.04	1.5±0.02^*^	2.0±0.04^**^	2.0±0.03	2.0±0.03	2.1±0.05
*Pythium ultimum* USB-F2	6.0±0.05	5.1±0.03^*^	5.3±0.03^*^	5.5±0.05^*^	5.3±0.04^*^	5.3±0.07^*^	5.3±0.05^*^
*Rhizoctonia solani* USB-F3	6.5±0.05	5.6±0.05^*^	5.6±0.03^*^	6.6±0.06	5.5±0.05^*^	6.5±0.04	6.4±0.06
*Rosellinia necatrix* USB-F587	5.5±0.04	3.1±0.03^*^	1.7±0.05^*^	5.4±0.03	3.1±0.04^*^	5.3±0.03	4.5±0.05^*^
*Sclerotinia sclerotiorum* USB-F593	5.6±0.04	2.7±0.07^*^	3.4±0.05^*^	5.5±0.06	3.4±0.05^*^	4.8±0.03^*^	3.4±0.03^*^
*S. sclerotiorum* USB-F853	3.6±0.04	1.6±0.05^*^	1.6±0.06^*^	3.6±0.03	1.6±0.04^*^	3.5±0.05	1.6±0.03^*^
*Verticillium dahliae* USB-F464	1.7±0.02	1.2±0.03^*^	1.5±0.03^*^	1.6±0.03^*^	1.1±0.03^*^	1.5±0.04^*^	1.6±0.03^*^
*V. dahliae* ITM-1910	1.7±0.03	1.1±0.03^*^	1.5±0.03^**^	1.6±0.03	1.0±0.02^*^	1.3±0.03^*^	1.4±0.03^*^

a Microorganisms were grown on KBA (King et al., 1954).

b Results are shown in centimeters ± standard error of mycelium growth.

c Control, fungus inoculated without bacteria; bacterial isolates attributable, on the basis of the partial 16SrDNA sequencing, to Pseudomonas brassicacearum (USB2101, USBB2102, and USB2104); Bacillus megaterium (USB2103) and Pseudomonas putida (USB2105 and USB2106).

* P ≤ 0.001.

** 0.001 < P < 0.05.

#### Volatile compounds

*In vitro* assays have shown that bacterial volatiles are able to inhibit fungal growth as well. In general, volatiles of all rhizobacteria highly inhibited the growth of almost all the target fungi used in this study when grown on KBA (Table 3). In particular, all the isolates, apart from USB2103 of *Bacillus* spp., showed strong fungal growth inhibition activity via volatiles (Table 3). The strains USB-F1131 and USB-F1636 of *B. cinerea*, USB-F172 of *P. nicotianae*, USB-F3 of *Rhizoctonia solani*, USB-F593 of *S. sclerotiorum* and ITM-1910 of *Verticillium dahliae* were the most inhibited fungi by bacterial volatiles. On the other side, the strains USB-F111 and USB-F1 of *F. oxysporum* and the strain USB-F918 of *Macrophomina phaseolina* resulted the less inhibited in their growth (Table 3), even though bacterial volatiles determined a loss of pigmentation of the three pathogenic fungi mycelia (data not shown). Among the most inhibited fungi the strain USB-F593 of *S. sclerotiorum* was selected for further studies to check bacterial volatiles toxic effect on fungal mycelium and sclerotia. However, the inhibition of fungal growth by bacterial volatiles on the strain USB-F593 of *S. sclerotiorum* resulted apparently fungistatic. Indeed, when plugs of mycelium were removed from PDA plates exposed to bacterial volatiles for 5 days, and placed on fresh PDA, the fungus was able seemingly to grow at the same development rate of the control plug, although the growth showed, by eye, a thinning of the mycelium compared to the fluffy mycelium of the control (Table 4).

**Table 3:** **Growth inhibition of phytopathogenic fungi exposed to bean rhizobacteria volatiles assessed by double plate technique**.

**Phytopathogenic fungal strains^a^**	**Treatments^a^^,^^b^^,^^c^**						
	**Control**	**USB2101**	**USB2102**	**USB2103**	**USB2104**	**USB2105**	**USB2106**
*Botrytis cinerea* USB-F1131	1.6±0.03	0.1±0.02^*^	0.1±0.03^*^	1.5±0.02	0.1±0.03^*^	0.1±0.02^*^	0.1±0.03^*^
*B. cinerea* USB-F1636	1.6±0.06	0.1±0.02^*^	0.1±0.03^*^	0.9±0.05^*^	0.1±0.03^*^	0.1±0.03^*^	0.1±0.02^*^
*Fusarum equiseti* USB-F2014	2.7±0.02	1.1±0.03^*^	0.9±0.02^*^	2.7±0.05	1.2±0.04^*^	0.9±0.03^*^	0.8±0.05^*^
*F. oxysporum* USB-F1	2.8±0.03	2.8±0.03	2.8±0.02	2.9±0.03	2.8±0.02	1.7±0.03^*^	2.0±0.03^*^
*F. oxysporum* USB-F111	5.8±0.05	5.0±0.03^*^	4.9±0.04^*^	5.8±0.03	5.0±0.03^*^	4.9±0.05^*^	4.9±0.03^*^
*F. solani* USB-F607	5.6±0.03	3.9±0.02^*^	3.3±0.03^*^	4.7±0.02^*^	3.6±0.03^*^	3.9±0.03^*^	3.9±0.05^*^
*Macrophomina phaseolina* USB-F918	5.9±0.05	4.2±0.05^*^	5.9±0.04	6.0±0.03	5.0±0.03^*^	5.0±0.05^*^	5.1±0.05^*^
*Phytophthora cactorum* USB-F876	1.8±0.03	1.8±0.02	0.6±0.04^*^	1.8±0.03	1.2±0.02^*^	1.2±0.05^*^	1.2±0.03^*^
*P. cactorum* USB-F1168	2.0±0.02	2.0±0.02	1.2±0.03^*^	1.9±0.02	1.1±0.05^*^	1.1±0.03^*^	1.1±0.04^*^
*P. nicotianae* USB-F172	2.2±0.07	0.2±0.02^*^	0.3±0.04^*^	2.1±0.04	0.3±0.02^*^	0.3±0.03^*^	0.2±0.03^*^
*Pythium ultimum* USB-F2	5.9±0.06	4.3±0.05^*^	4.9±0.03^*^	5.0±0.05^*^	3.5±0.03^*^	0^*^	0^*^
*Rhizoctonia solani* USB-F3	6.5±0.05	0.5±0.03^*^	0.5±0.04^*^	3.5±0.05^*^	0.3±0.05^*^	0.5±0.02^*^	3.5±0.05^*^
*Rosellinia necatrix* USB-F587	5.4±0.03	4.3±0.11^*^	0.7±0.03^*^	5.4±0.04	2.2±0.03^*^	0.7±0.05^*^	3.5±0.03^*^
*Sclerotinia sclerotiorum* USB-F593	5.6±0.05	0^*^	0^*^	4.4±0.03^*^	0^*^	0.6±0.05^*^	0.5±0.03^*^
*S. sclerotiorum* USB-F853	3.6±0.04	0^*^	0^*^	3.6±0.05	0^*^	2.8±0.03^*^	2.3±0.05^*^
*Verticillium dahliae* USB-F464	1.8±0.03	1.9±0.04	0.4±0.03^*^	1.4±0.03^*^	0.9±0.03^*^	1.4±0.05^*^	0.4±0.03^*^
*V. dahliae* ITM-1910	1.7±0.03	0^*^	0^*^	0.9±0.02^*^	0^*^	0^*^	1.0±0.02^*^

a Bacteria were grown on KBA (King et al., 1954), while fungi on PDA.

b Results are shown in centimeters ± standard error of mycelium growth.

c Control, fungus inoculated without bacteria; bacterial isolates attributable, on the basis of the partial 16SrDNA sequencing, to Pseudomonas brassicacearum (USB2101, USBB2102, and USB2104); Bacillus megaterium (USB2103) and Pseudomonas putida (USB2105 and USB2106).

* P ≤ 0.001.

**Table 4:** **Mycelia growth of ***Sclerotinia sclerotiorum*** USB-F593 from fungal plug and sclerotia after 5 days exposure to rhizobacteria volatiles and from fungal plug and sclerotia re-inoculated on fresh PDA**.

**Treatments^a^**	**Mycelium diameters (cm)^b^**			
	**VOCs assay on mycelium**	**Viability assay of mycelium**	**VOCs assay on sclerotia**	**Viability assay of sclerotia**
Control	5.6 ± 0.05	5.8 ± 0.08	3.2 ± 0.08	3.5 ± 0.06
USB2101	0^*^	5.7 ± 0.08	0^*^	3.4 ± 0.04
USB2102	0^*^	5.8 ± 0.07	0^*^	3.4 ± 0.06
USB2103	4.4 ± 0.03^*^	5.9 ± 0.04	2.6 ± 0.06^*^	3.7 ± 0.06
USB2104	0^*^	5.9 ± 0.05	0^*^	3.4 ± 0.05
USB2105	0.6 ± 0.05^*^	5.7 ± 0.03	0.3 ± 0.03^*^	3.4 ± 0.06
USB2106	0.5 ± 0.03^*^	5.7 ± 0.05	0.2 ± 0.03^*^	3.5 ± 0.05

a Control = fungus inoculated without bacteria; bacterial isolates attributable, on the basis of the partial 16SrDNA sequencing, to Pseudomonas brassicacearum (USB2101, USBB2102, and USB2104); Bacillus megaterium (USB2103) and Pseudomonas putida (USB2105 and USB2106).

a Results are shown in centimeters ± standard error of mycelium growth.

* P ≤ 0.001.

#### Sclerotia viability assays

Bacterial volatiles caused the total lack of germination of sclerotia in the treatments with the *Pseudomonas* spp. isolates USB2101, USB2102, and USB2104; on the contrary, mycelium growth from sclerotia was reduced of about 80% by volatiles of the *Pseudomonas* spp. isolates USB2105 and USB2106 and about 40% by the isolate USB2103 of *Bacillus* spp. (Table 4). Moreover, after treatments with rhizobacteria volatiles, when re-inoculated on fresh PDA, sclerotia germinated forming mycelium at the same radial growth rate than the control (Table 4), even though thinner and not fluffy as the control, confirming the apparent fungistatic nature of bacterial volatiles.

### GC-MS analysis

The results of the qualitative GC-MS analysis of the VOCs produced by rhizobacteria after 5 days incubation are listed in Table 5. Time course GC-MS analysis revealed that bacteria under study produce VOCs among which several ones were found in every time points, while other VOCs were detected in a specific time point. In other words, bacteria produce a typical volatiles blend depending on their growth stage. VOCs identified at the three time point considered are listed in Table 6.

**Table 5:** **Volatile organic compounds (VOCs) produced by rhizobacteria and detected by GC-MS analysis after 5 days incubation**.

**VOCs^a^**	**Producers^b^**
acetic acid	USB2104
m-cymene	USB2104
dimethyl disulfide	USB2105, USB2106
dimethyl trisulfide	USB2105, USB2106
dl-limonene	USB2104
2-nonanone	USB2101, USBB2102, USB2104, USB2105
2-propanone	USB2103
1-tetradecanol	USB2103
2-undecanone	USB2101, USBB2102, USB2104, USB2105
1-undecene	USB2101, USBB2102, USB2104, USB2105

a VOCs identified with a score > 80%.

b Bacterial isolates attributable, on the basis of the partial 16SrDNA sequencing, to Pseudomonas brassicacearum (USB2101, USBB2102, and USB2104); Bacillus megaterium (USB2103) and Pseudomonas putida (USB2105 and USB2106).

**Table 6:** **Volatile organic compounds (VOCs) time-course profiles produced by selected rhizobacteria isolated from common bean as determined by GC-MS analysis**.

**VOCs**	**Bacterial isolates^a^^,^^b^**								
	**USB2103**			**USB2104**			**USB2105**		
	**1**	**3**	**5**	**1**	**3**	**5**	**1**	**3**	**5**
acetic acid						x			
2-bromo-dodecane		x			x		x		
2-bromo-tetradecane	x								
4-butyl-cicloexene								x	
m-cymene						x			
3-decen-1-ol acetate				x	x				
3,6- dimethyl-decane	x			x	x			x	
dimethyl disulfide							x	x	x
dimethyl trisulfide									x
4,7-dimethyl-undecane		x			x				
dl-limonene						x			
eicosane		x					x		
2-ethyl-2-methyl-tridecanol		x							
5-ethyl-2-methyl-octane	x								
heneicosane	x	x		x	x		x	x	
hentriacontane				x					
heptacosane	x	x		x	x		x	x	
heptadecane	x			x			x		
hexacosane	x							x	
hexadecane	x							x	
1-iodo-dodecane					x				
1-iodo-octadecane	x						x		
1-iodo-tridecane								x	
methanethiol							X		
2-methyl-heptadecane	x						x		
8-methyl-heptadecane	x						x	x	
4-methyl-tetradecane	x				x				
2-nonanone						x		x	x
octacosane	x	x		x	x		x	x	
octadecane				x					
pentacosane	X			x			x		
pentadecane					x			x	
2-pentadecanone					x				
2-propanone			x						
1-tetradecanol			x						
triacontane				x			x		
2-tridecanone					x			x	
2,3,7-trimethyl-decane					x				
2,6,11-trimethyl-dodecane					x			x	
2-undecanone				x	x	x		x	x
1-undecene				x	x	x	x	x	x

a Bacterial isolates attributable, on the basis of the partial 16SrDNA sequencing, to Bacillus megaterium (USB2103); Pseudomonas brassicacearum (USB2104) and Pseudomonas putida (USB2105).

b Time points considered for the analysis: 1, 3, and 5 days of bacteria incubation.

Among the VOCs identified, 1-undecene, 2-nonanone, 2-undecanone, 2-propanone, 1-tetradecanol, acetic acid, m-cymene, dl-limonene, dimethyl disulfide, and dimethyl trisulfide were selected in order to assess their single biological activity. The selection was based on their detection at the 5^th^ day of bacterial incubation, on their systematic detection in all the three times that the analysis was performed, on the concentration of the components evaluated as peak area and their availability on the market.

### Effects of pure VOCs on *Sclerotinia sclerotiorum* mycelium and sclerotia

The results from assays performed using pure VOCs toward strain USB-F593 of *S. sclerotiorum* are showed in Table 7.

**Table 7:** **Antifungal activity toward ***Sclerotinia sclerotiorum*** strain USB-F593 mycelium and sclerotia, and hemolytic activity of representative volatile organic compounds produced by the six rhizobacteria isolated from common bean**.

**VOCs**	**MIQ on mycelium**	**MIQ on sclerotia**	**MHQ**
acetic acid	4.19	9.44	0.066
m-cymene	13.77	17.22	0.215
dimethyl disulfide	31.38	73.22	0.065
dimethyl trisulfide	24.04	24.04	0.075
dl-limonene	17.20	30.10	0.215
2-nonanone	4.92	16.4	0.102
2-propanone	NA	NA	79.1
1-tetradecanol	NA	NA	NA
2-undecanone	14.85	16.5	0.206
1-undecene	NA	NA	75.1

MIQ, Minimal Inhibitory Quantity, expressed in milligrams, able to reduce mycelium growth and sclerotia germination. MHQ, Minimal Hemolytic Quantity, expressed in milligrams, able to lyse red blood cells. MIQ and MHQ were calculated considering the applied volumes and the densities of each compound. The values reported stand for the average of the values from three experiments. NA, No detected activity applying 100 μl of pure compounds.

The acetic acid and 2-nonanone, with MIQs of 4.19 and 4.92 mg, respectively, resulted the most active compounds in reducing mycelium growth arising from the fungal plug (Table 7). On the contrary, 2-propanone, 1-tetradecanol, and 1-undecene appeared to be completely inactive in inhibiting fungal growth at all the quantities applied (Table 7). Higher MIQ with values comprised between 13.77 and 31.38 mg were observed for the other VOCs (Table 7).

A similar effects trend was observed in the sclerotia germination assay, though the MIQs were higher than those observed for the mycelium growth. An exception was observed for dimethyl trisulfide whose MIQ resulted similar either on mycelium growth or sclerotia germination (Table 7).

### Hemolytic activity of pure VOCs

All the pure VOCs used in this work, apart from 1-tetradecanol, were able to lyse red blood cells (Table 7). Dimethyl disulfide and acetic acid resulted the most active since they showed MHQs of 0.065 and 0.066 mg, respectively (Table 7). On the contrary, 1-undecene and 2-propanone showed hemolytic activity with MHQ of 75.1 and 79 mg, respectively (Table 7).

### Microsopic observations and ultrastructural studies

**Figure 1:**
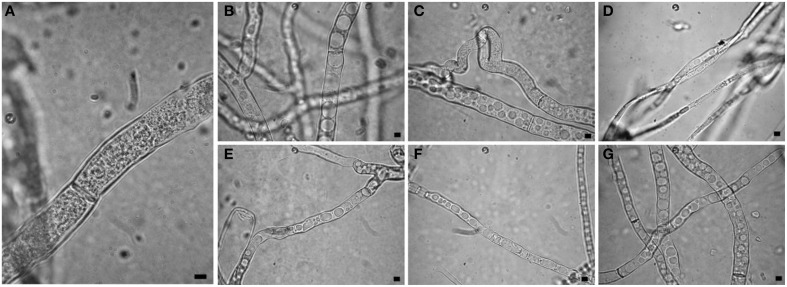
**Light observations of ***Sclerotinia sclerotiorum*** hyphae exposed to bacterial volatiles**. **(A)** control; **(B,C,E–G)** strains USB2101, USB2102, USB2104, USB2105, and USB2106 of *Pseudomonas* spp.; **(D)** strain USB2103 of *Bacillus* spp. Bars = 2 μm.

**Figure 2:**
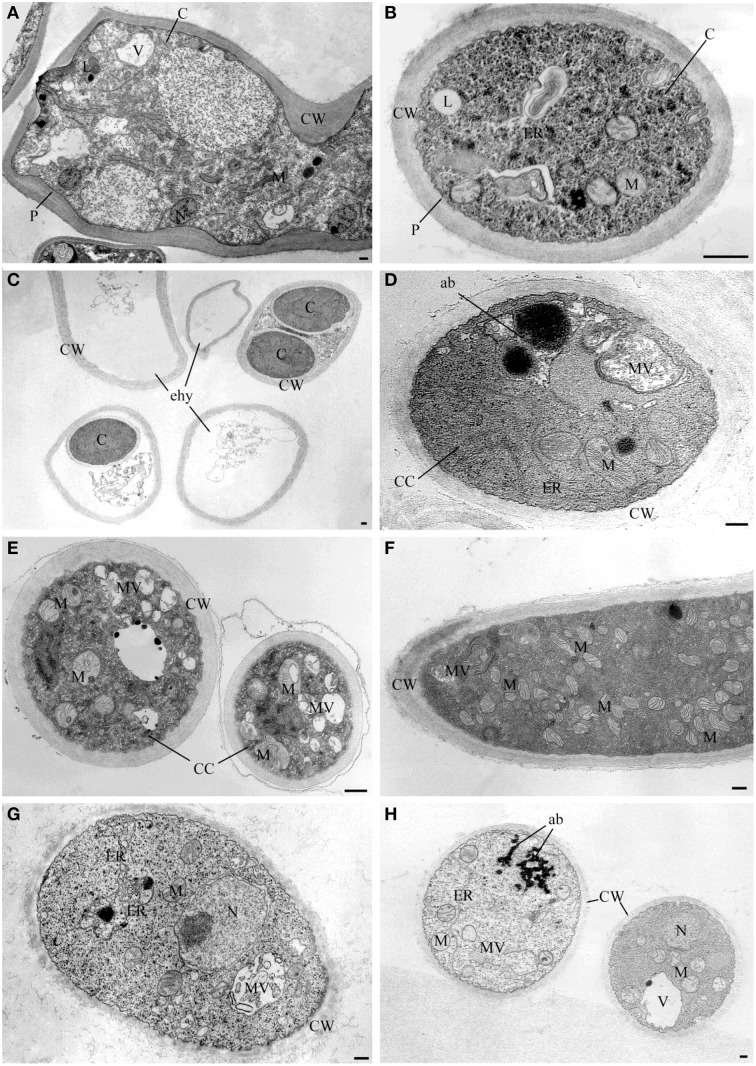
**TEM observations of ***Sclerotinia sclerotiorum*** hyphae exposed to bacterial volatiles. (A,B)** TEM micrographs showing hyphae ultrastructures of the strain USB-F593 *S. sclerotiorum* mycelium not exposed to bacterial volatiles. TEM micrographs showing ultrastructural changes of *S. sclerotiorum* USB-F593 mycelium exposed to volatiles of the *Pseudomonas* spp. USB2104 **(C,D)**, USB2105 **(E,F)** and *Bacillus* spp. USB2103 **(G,H)**. ab, accumulation body; C, cytoplasm; CC, condensed cytoplasm; CW, cell wall; ER, endoplasmic reticulum; ehy, empty hyphae; M, mitochondria; MV, multivesicular; N, nucleus; P, plasmalemma. Bars: 250 nm.

Observations at light microscope of the strain USB-F593 of *S. sclerotiorum* mycelium, exposed to bacterial volatiles of the six rhizobacteria, showed hyphae morphological abnormalities compared to the control treatment. In fact, in all the treatments, hyphae, observed by optical light microscope, appeared thinner and characterized by the presence of vacuolization of cytoplasm when compared to the control (Figure 1). Further observations at TEM of the mycelium treated with bacterial volatiles showed the alterations of the hyphae ultra-structures confirming the hyphae morphology alteration observed by light microscopy. In particular, thin sections of the *S. sclerotiorum* mycelium not exposed to bacterial volatiles, observed at TEM, revealed characteristic ascomycete hyphae ultra-structures. Indeed, a typical septum showing a single hole, normo-functional cytoplasm, good adhesion between the cytoplasmic membrane and the outer wall, various organelles, distinct, and normal in their constituent elements, normal size vacuoles, regularly shaped mitochondria and portions of rough endoplasmic reticulum were observed (Figures 2A,B). In the samples exposed to volatiles of the *Pseudomonas* spp. isolate USB2104 some hyphae showed very condensed cytoplasm, others were empty, in some other cases hyphae appeared duplicated and were apparently included in wall-like structures. It was also noticed cytoplasmic membrane detachment from the cell wall, numerous and hyper-crested mitochondria, multi-vesiculation, cytoplasmic accumulations of material (protein or lipid), hypertrophy of the endoplasmic reticulum (Figures 2C,D). Volatiles of *Pseudomonas* spp. isolate USB2105 caused the thickening of the hyphae cell wall and hyphae showed very condensed and granulated cytoplasm. Furthermore, hyphae presented numerous mitochondria with iper-vesiculated and hypertrophic ridges and multi-vesiculation and cytoplasmic accumulation of material (protein or lipid) (Figures 2E,F). The exposure to volatiles of *Bacillus* spp. isolate USB2103 led to cytoplasm thickening, to the partial detachment of outer nuclear membrane, to hypo-crested mitochondria with denser matrix as well as accumulation of lipids and proteins in the cytoplasm and into the endoplasmic reticulum which also showed hyperplasia (Figures 2G,H).

**Figure 3:**
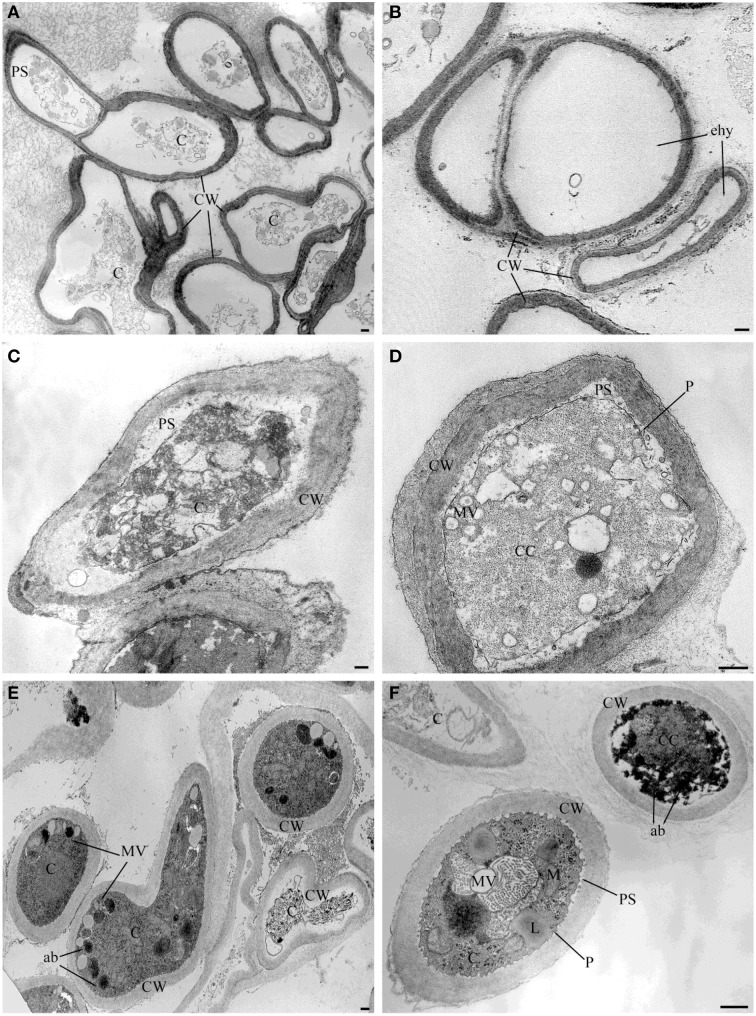
**TEM observations of ***Sclerotinia sclerotiorum*** hyphae exposed to pure VOCs**. Ultrastructural changes of the strain USB-F593 of *S. sclerotiorum* mycelium treated with pure VOCs 2-nonanone **(A,B)**, dl-limonene **(C,D)** and dimethyl disulfide **(E,F)**. ab, accumulation body; C, cytoplasm; CC, condensed cytoplasm; CW, cell wall; ehy, empty hyphae; MV, multivesicular; P, plasmalemma: PS, periplasmic space. Bars: 250 nm.

TEM observation of the samples treated with the pure VOCs 2-nonanone, dl-limonene and dimethyl disulfide applied at their MIQ confirmed their involvement in the alteration of hyphae ultrastructures noticed in the samples exposed to the natural blend of bacterial volatiles. In particular, 2-nonanone caused complete or partial hyphae emptying due to the damage of cytoplasmic membrane which, in fact, resulted detached from the outer wall; strong vacuolization with internal residues of membranes and cytoplasmic matrix in the cytoplasm were also noticed (Figures 3A,B). dl-Limonene treatment led to granulation of hyphae cytoplasm, cytoplasmic membrane detachment from the cell wall that resulted thickened; it was also noticed absence of organelles, multi-vesciculation and accumulations of proteic and lipidic material in the cytoplasm (Figures 3C,D). Lastly, dimethyl disulfide treated samples showed strongly marked ultrastructural modification: hyphae mostly appeared with missing or altered cytoplasm, hyper-vesiculation, hypocrested and vesiculated mitochondria which resulted fewer than the control, and accumulations of proteic and lipidic material in the cytoplasm (Figures 3E,F). Nevertheless, in the all TEM treated samples apparently normal hyphae were also observed, even though very few compared to the damaged ones.

## Discussion

Recent our studies showed the potential ability of six rhizobacteria, isolated from bean plants, to control *in vitro* and greenhouse CBB and to have several typical important phenotypic traits of biocontrol agents (Giorgio et al., 2015) (Supplementary Table 1). In this study the above rhizobacteria were evaluated for their capability to inhibit *in vitro* the growth of several phytopathogenic fungi, mostly soil-borne pathogens, for their possible use as antagonists in fungal disease protection of bean as well as other horticulture crops. The six rhizobacteria strongly affected fungal growth, though a different sensitivity among the phytopathogenic fungi was observed, not only via direct diffusible substances but also via volatiles-mediated action. In this work particular emphasis has been given to bacterial volatiles effects since they may play a pivotal role in the interaction between organisms living the same ecological niche (Popova et al., 2014). Among the phytopathogenic soil borne fungi screened in this study, the strain USB-F593 of *S. sclerotiorum* isolated from bean, resulted mostly affected by bacterial volatiles and for that selected for further studies. Furthermore, of interest is the fact that in the volatiles assays a loss of pigmentation in *F. oxysporum* and *M. phaseolina* strains was also noticed. The alteration observed could play an important significance not only for metabolic aspects of the fungi but also for some features related to the pathogen virulence/pathogenicity as well as to the reduction/loss of its antimicrobial weapon arsenal. Indeed, the observed loss of melanin by *M. phaseolina* may negatively influence survival, pathogenicity, and recovery functions of the pathogen from radiation and oxidizing agents (Dhingra and Sinclair, 1978; El Bassam et al., 2002; Cao et al., 2006). Similarly, pinkish-purple naphthoquinones produced by *F. oxysporum* were demonstrated to have antimicrobial activity (Visconti et al., 1983; Baker et al., 1990; Medentsev and Akimenko, 1998).

Observations with light microscope of *S. sclerotiorum* mycelium, exposed to the six rhizobacteria volatile blends, revealed thinner hyphae characterized by the presence of vacuolization in the cytoplasm, compared to control, indicating cytoplasmic membrane as a possible target of the volatile mixtures. TEM observations of *S. sclerotiorum* mycelium exposed to three out the six rhizobacteria (namely USB2103, USB2104, USB2105) confirmed the hyphae ultrastructural alterations due to the loss of cell membranes integrity and the consequent cell permeability alteration, as confirmed by the hemolytic activity shown by most of the pure VOCs. Furthermore, the alteration of mitochondrial membranes confirmed the membrane system as one of volatiles target. To our knowledge, this is the first work in which the individual activity of bacterial VOCs on phyto-pathogenic fungi is investigated at the ultrastructural level. Moreover, in the case of *Pseudomonas* spp. isolate USB2105 volatiles exposure a proliferation of mitochondria or mitochondria cristae hypertrophy was noticed indicating possible increased respiratory requirements as a result of bacterial volatiles toxic action.

GC-MS analysis of bacterial volatiles, after 5 days incubation, showed some similarity of the volatile profiles of the five isolates of *Pseudomonas* spp. under study; indeed four out the five isolates (USB2101, USB2102, USB2105, USB2106) produced 1-undecene, 2-nonanone, and 2-undecanone. However, the *Pseudomonas* spp. isolate USB2104 produced in addition acetic acid, m-cymene and dl-limonene. 1-Undecene is a terminal olefin demonstrated to have strong toxic effects on *Drosophila melanogaster* and the nematode *Caernorhabditis elegans* but no effect on bacteria or pathogenic fungi growth has been reported (Popova et al., 2014). In fact, even in this study, this substance showed no apparent effect on fungal growth. On the contrary, the methyl ketones 2-nonanone and 2-undecanone, coming from the same metabolic pathway (Park et al., 2012), showed strong fungal growth inhibition. Methyl ketones, commonly found in nature as constituent of essential oils of several plants and produced by bacteria, fungi, insects (Cavill et al., 1956; Cavill and Hinterberger, 1960; Bernardi et al., 1967; Moser et al., 1968), and mammalian cells (Forney and Markovetz, 1971), present a variety of biological properties and potential commercial exploitations (Forney and Markovetz, 1971; Bolster et al., 2001; Antonious et al., 2003; Guo et al., 2008; Shi et al., 2008; Zhu and Hua, 2009). For example, 2-nonanone, released by red raspberries and strawberries during ripening, was demonstrated to inhibit postharvest decay fungi in strawberry fruit (Vaughn et al., 1993) and both 2-nonanone and 2-undecanone from soil bacteria have nematicidal action (Gu et al., 2007). The activity of such molecules is related to their ability to cross lipid layers of cell membranes in relation to their lipophilicity which depends on the acyl chain length appearing optimal when is 9–14 carbons long (Dimock et al., 1982). In our hemolytic experiments pure VOCs 2-nonanone and 2-undecanone demonstrated to lyse red blood cells confirming their action on biological membranes; ultrastructural hyphae alterations at the membranes level was confirmed in TEM observations of mycelium treated with 2-nonanone. Furthermore, the observed fungal cell wall darkening, probably due to chitin condensation, suggests that this ketone may alter chitin chemical structure possibly via nucleophilic addition between its carbonyl group and the acetyl amine groups of fungal chitin (Carey, 2003). Acetic acid, m-cymene and dl-limonene inhibited fungal growth and showed hemolytic activity as well stating their action on cell membranes. Acetic acid is a natural compound found throughout the biosphere and has long been known for its flavoring and preservative properties against microorganisms in a variety of food products (Davidson et al., 2002; Alawlaqi and Alharbi, 2014). Its anti-microbial activity is exerted by the capacity to penetrate the microbial cell interfering with transport of metabolites and maintenance of potential at membrane level (Banwart, 1981). m-Cymene is a benzene derivative terpene, constituent of a number of essential oils such as cumin and thyme, whose antimicrobial properties were noticed for the first time by Prasad et al. (2010). The other terpene detected, dl-limonene, is more common in nature as the main constituent of citrus essential oil and it has antifungal (Chee et al., 2009) but limited antibacterial activities (Lo Cantore et al., 2009) and extremely wide industrial applications (Pakdel et al., 1991; Dambolena et al., 2008). In the present work TEM observations showed visible ultrastructural damage of *S. sclerotiorum* hyphae apparently due to limonene toxic action (Chee et al., 2009).

The shorter chain methyl ketone 2-propanone, detected in the volatiles natural blend of the isolate USB2103 of *Bacillus* spp., did not apparently affect the fungal growth when tested alone confirming the previously observed low toxicity. Isolate USB2103 resulted to produce 1-tetradecanol as well which did not show fungal growth inhibitory activity; this is not surprising since the compound is generally considered having low toxicity (Wigaeus et al., 1981; Gorsuch et al., 1990; Hernandez, 1999) because of its limited amphipathic properties that lead to low interference with membranes.

The isolates USB2105 and USB2106 of *Pseudomonas* spp. produced, besides the above mentioned volatiles, dimethyl disulfide and dimethyl trisulfide, that exhibited antifungal action on *S. sclerotiorum* mycelium and inhibited sclerotia germination according to literature data (Fernando et al., 2005; Kai et al., 2009). The bacterial VOCs action on sclerotia germination is important since the control and eradication of this pathogen is difficult due to the environmental resistance of these structures that can survive in soil for years without hosts or favorable condition for development (Coley-Smith and Cooke, 1971). It has been suggested that dimethyl disulfide and dimethyl trisulfide play a role in plant protection, acting in the control of plant-pathogenic fungi (Kai et al., 2009), weeds (Freeman et al., 2009), and nematodes (Coosemans, 2005). For the first time here the hemolytic activity of the above substances has been unraveled. TEM observations of dimethyl disulfide treated mycelium revealed a high damage on organelles and the cytoplasmic organization. According to literature data, dimethyl disulfide is a small molecule capable to cross over the cell wall and membranes and it was demonstrated that, in different organisms, it acts as a powerful inhibitor of complex IV of cytochrome oxidase in mitochondria (Dugravot et al., 2003). This malfunction may contribute to explain the decrease of mitochondria number that appeared hypocrested and vesiculated in our TEM observations.

Even though volatiles natural blend of isolate USB2103 of *Bacillus* spp. affected to a lesser extent *S. sclerotiorum* mycelia growth, TEM observations noticed structural damages suggest possible synergy between the various constituents of natural volatile mixture. Moreover, it is not to be excluded that other bioactive volatiles were, for some reasons, not detected in GC-MS analysis condition.

Summarizing, it can be stated that individual VOCs showed, at least for most of them, the involvement in growth suppression of *S. sclerotiorum* and the pathogen membranes are one of their targets, as suggested by their hemolytic activities and membranes alteration, beside other observed ultrastructural changes on cell organelles such as mitochondria and endoplasmic reticulum.

The apparent fungistatic action of VOCs, as suggested by the fact that mycelium plugs of *S. sclerotiorum* radial grow at a rate similar to the control when transferred on fresh PDA, is in apparent contradiction with the observed alterations on hyphae morphology and cell ultrastructures. This can be explained by the fact that in the mycelium masses some hyphae not altered yet and alive, as observed in TEM analysis, are present. This may be due to differences in the hyphae response to the VOCs exposure in relation to hyphae different physiological state. Similar consideration may be done in the case of not germinated sclerotia after exposure to bacterial volatiles. Nevertheless, the VOCs appears good candidates for the control of this soil fungal pathogen as fumigants.

Apart from the inhibitory activity that bacterial VOCs could have to prevent the development of fungal mycelium, some other volatiles can contribute to the observed effects, being an integrative part of the pool of biologically active volatiles produced by the tested bacteria. This is the case of hydrogen cyanide or ammonia produced by the three *Pseudomonas* spp. attributable to *P. brassicacearum* and the *P. putida* likely strains. Although our study was mostly focused on the investigation on the effects of VOCs detected and identified after 5 days incubation, it is not to be excluded that the final effects on fungal structures may be the outcome of the action of all the volatiles produced along the whole incubation period. Indeed, in our GC-MS time course analysis some other VOCs were detected and this finding deserves to be deepened in future studies.

### Conflict of interest statement

The authors declare that the research was conducted in the absence of any commercial or financial relationships that could be construed as a potential conflict of interest.
