# Supplementary material for: Biocide effects of volatile organic compounds produced by potential biocontrol rhizobacteria on Sclerotinia sclerotiorum
Source: Front Microbiol. 2015 Oct 6;6:1056. doi: 10.3389/fmicb.2015.01056 (PMC4594563; doi:10.3389/fmicb.2015.01056)
Supplement: Supplementary file 1 [file Table1.DOC]

**Supplementary Table 1.** Phenotypic characters of bacteria isolated from bean rhizosphere.

| **Phenotype features** | | **Bacterial isolates^a^** | | | | | |
| --- | --- | --- | --- | --- | --- | --- | --- |
|  |  | **USB2101** | **USB2102** | **USB2103** | **USB2104** | **USB2105** | **USB2106** |
| Gram staining | | - | - | + | - | - | - |
| Fluorescence | | + | + | - | + | + | + |
| Hydrolytic enzymes produced | Pectinase | + | + | + | + | + | + |
|  | Cellulase | - | + | - | + | - | - |
|  | Protease | - | - | + | + | + | + |
|  | Amylase | - | - | - | - | + | + |
|  | Gluganase | - | - | + | + | + | + |
|  | Chitinase | - | - | - | - | - | - |
| Phosphate solubilization | | + | + | + | + | + | + |
| Indole compounds production | | + | + | + | + | + | + |
| Nitrogen fixing activity | | - | - | - | - | - | - |
| Siderophores production | | + | + | + | + | + | + |
| Haemolysis | | + | + | + | + | + | + |
| HCN production | | + | + | - | + | - | - |
| Ammonia production | | - | - | - | - | + | + |
| NaCl  tolerance  (%) | 0.5 | + | + | + | + | + | + |
|  | 1.0 | + | + | + | + | + | + |
|  | 5.0 | + | + | + | + | + | + |
|  | 10.0 | - | - | + | - | + | + |
|  | 15.0 | - | - | + | - | + | + |
|  | 20.0 | - | - | + | - | - | - |
| pH variation resistance | 4.0 | - | + | - | + | + | - |
|  | 7.0 | + | + | + | + | + | + |
|  | 10.0 | - | + | + | - | + | + |
| T variation resistance (°C) | 10.0 | + | + | + | + | + | + |
|  | 25.0 | + | + | + | + | + | + |
|  | 60.0 | - | - | + | - | - | - |

^a^Bacterial isolates attributable, on the basis of the partial 16SrDNA sequencing, to *Pseudomonas brassicacearum* (USB2101, USB2102 and USB2104); *Bacillus megaterium* (USB2103) and *P. putida* (USB2105 and USB2106).
